# Psychotic disorders in HIV-positive versus HIV-negative patients: comparative study of clinical characteristics

**DOI:** 10.1192/bjo.2024.771

**Published:** 2024-12-13

**Authors:** Noeline Nakasujja, Seggane Musisi, Hans Agren, Elly Katabira, Peter Allebeck

**Affiliations:** Department of Psychiatry, College of Health Sciences, Makerere University, Kampala, Uganda; and Department of Public Health Sciences, Karolinska Institutet, Stockholm, Sweden; Department of Psychiatry, College of Health Sciences, Makerere University, Kampala, Uganda; Department of Psychiatry and Neurochemistry, Institute of Neuroscience and Physiology, University of Gothenburg, Gothenburg, Sweden; Department of Medicine Makerere, University College of Health Sciences, Kampala, Uganda; Department of Public Health Sciences, Karolinska Institutet, Stockholm, Sweden

**Keywords:** HIV, AIDS, psychosis

## Abstract

**Background:**

Clinical characteristics of psychosis in HIV infection have been described, but there have been limited comparative studies in HIV-endemic low-resource regions.

**Aim:**

To compare clinical characteristics of psychosis in HIV-positive and HIV-negative patients at the main psychiatric referral units in Uganda.

**Method:**

Patients with psychosis were consecutively recruited and completed a standardised demographic questionnaire and psychiatric and laboratory assessments including an HIV test. The Mini International Neuropsychiatric Interview was used to diagnose psychiatric illness. Psychosis symptoms were compared between HIV-positive and HIV-negative individuals using bivariate methods. A logistic regression model was used to assess the effects of age, gender and HIV status on different types of psychosis.

**Results:**

There were 478 patients enrolled, of which 156 were HIV positive and 322 were HIV negative. The mean age was 33.2 years (95% CI 31.8–34.5) for the HIV-positive group and 29.6 years (95% CI 28.7–30.5) for the HIV-negative group (*P* < 0.001). Female patients had a higher proportion of seropositivity 40.6% (95% CI 34.8–46.4) compared with males 21.8% (95% CI 16.1–27.5) (*P* < 0.001). Psychotic disorder not otherwise specified occurred more in the HIV-positive individuals (88% (95% CI 82.9–93.1) *v.* 12% (95% CI 8.4–15.5), *P* < 0.001). Motor activity, irritability, emotional withdrawal, feelings of guilt, mannerisms and posturing, grandiosity, suspiciousness, unusual thoughts, blunted affect, excitement and disorientation were associated with HIV seropositivity.

**Conclusion:**

The presentation of psychosis in patients with HIV is unique to this HIV endemic setting. Characterisation of the symptomatology of patients presenting with psychosis is important for proper diagnosis and care.

Rates of first-episode psychosis have been shown to be higher in HIV-positive individuals compared with the general population.^1,2^ Moreover, the presentation and clinical characteristics of HIV-associated psychosis have been found to be different from those of primary psychoses in studies conducted on small populations.^2–5^ The presence of delusions or hallucinations in an individual with or without disorganised behaviour constitutes a psychotic state.^6^ This represents a significant mental disorder that substantially impairs functioning and influences thought processes, perception and cognitive abilities.^3^ Psychotic disorders can be categorised as either primary (for instance, schizophrenia or schizoaffective disorder) or secondary (for example, psychosis stemming from a medical condition such as HIV infection) conditions.^6^ At the onset of the HIV/AIDS epidemic, it emerged that some individuals developed psychosis in association with HIV infection.^4,7^ Although at the time there were still schools of thought that believed the psychoses that these individuals presented with were coincidental cases of schizophrenia or mania,^8^ it has become increasingly clear that the HIV virus triggers psychosis either directly or indirectly through opportunistic infections or as a consequence of medications used in HIV treatment.^9,10^ During the initial stages of the HIV epidemic, psychiatric symptoms were predominantly observed in men;^4^ however, subsequent evidence has shown that these manifestations affect individuals of both sexes.^11^

Psychosis is prevalent in individuals living with HIV in sub-Saharan Africa, particularly among those who may not have commenced antiretroviral therapy.^12^ Based on research conducted among HIV-positive individuals experiencing mania in Uganda, in comparison with the broader HIV-negative population with primary mania, HIV-positive patients appeared to be of more advanced age, with CD4 counts below 350 cells/mm^3^, exhibited more cognitive impairment, had lower levels of education and were more frequently female.^13,14^

The presentation of psychosis related to HIV tends to differ somewhat from that of primary psychosis. Harris et al^15^ identified persecutory, grandiose and somatic delusions as the most prevalent symptoms in new-onset psychosis in individuals with HIV disease, often accompanied by hallucinations and affective disturbances in more advanced stages of the illness. Conversely, De Ronchi et al^2^ proposed that paranoid delusions, in the absence of the usual affective symptoms, may constitute an ‘elementary model’ of acute psychosis in HIV patients, which sets it apart from primary psychosis. Unlike primary psychosis, which typically includes various mood changes such as anxiety, depression, mood swings, sleep disturbances, irritability, anger and suicidal ideation during its prodromal phase,^16^ patients living with HIV have been reported to exhibit more pronounced manic symptoms; they displayed increased irritability, greater aggression, heightened verbosity, and experienced a higher prevalence of paranoid delusions, visual hallucinations and auditory hallucinations.^13^

The World Health Organization (WHO) has developed a screening tool for staging HIV disease progression in resource-poor settings as a guide for triaging patients into care.^17^ In this tool, psychosis is identified as a late symptom of HIV presentation. Patients who experience the onset of mania during the advanced stages of HIV illness (late onset) exhibit a higher degree of manic symptoms. This suggests that patients with late-onset HIV mania present with more intense psychopathological symptoms and, consequently, warrant stronger consideration for highly active antiretroviral therapy.^17^

With a failure to perform the basic activities of daily living in patients that have psychosis,^18^ antiretroviral therapy is often stopped or never initiated for fear of the development of drug resistance.^19^ However, this raises a treatment quagmire in the management of HIV-related psychosis, especially when there is existing evidence indicating that antiretroviral therapy effectively mitigates psychotic symptoms.^20^ These and many challenges cause treatment delays for patients that have HIV and psychosis, thereby creating delays in the way health workers make care management decisions, some of them only based on their personal opinions.

The burden of HIV infection and its associated complications continues to be significant in Sub-Saharan Africa, with 10.3 million treatment-naive individuals and an estimated 760 000 new infections occurring annually in the region and with many of them still presenting late for HIV care.^21^ Though there have been a few studies on psychosis and HIV in the African setting,^13,22^ to our knowledge, there hasn't been any conducted on a substantial population to compare the clinical features of different types of psychosis between individuals who are HIV positive and those who are HIV negative. Understanding the clinical presentations of psychosis will help improve clinical care, hence this study that compared characteristics of psychosis using standard assessment instruments among HIV positive and HIV negative individuals.

## Method

We enrolled patients consecutively at Mulago and Butabika national referral hospitals in Kampala, Uganda. Research assistants approached all patients in recovery units and assessed them with respect to the inclusion and exclusion criteria.

### Inclusion criteria

Individuals were included in the study if they:

- had features of psychosis; i.e. had hallucinations, delusions, disorganised speech or behaviour, or catatonia;
- were aged 18–59 years;
- were resident within a radius of 30 km from the city centre;
- gave written informed consent to participate in the study.

### Exclusion criteria

Individuals were excluded from the study if they:

- could not speak English or Luganda;
- were too ill to withstand the evaluation 2 weeks after admission.

Nearly all patients who were approached consented to participate in the study, with the exception of two who refused consent.

### Sample size estimation

The sample size was calculated based on a study by Maling et al^23^ that found an HIV prevalence of 18.4% among individuals with first-episode psychosis. The ratio of HIV-negative to HIV-positive participants was predetermined as 2:1 using Bland's formula for comparing proportions.^24^

### Ethical considerations

The authors assert that all procedures contributing to this work comply with the ethical standards of the relevant national and institutional committees on human experimentation and with the Helsinki Declaration of 1975, as revised in 2008. All procedures involving human subjects/patients were approved by Makerere University Research and Ethics committee as well as the Uganda National Council for Science and Technology- HS #385. The research assistants obtained consent from willing participants once they were calm or strong enough to withstand the interview procedures. Written consent and the interview typically took place approximately 1–2 weeks from the time of admission. Any individuals who declined to participate further received clinical care in the hospital without prejudice. The research interviews primarily took place in the native language, Luganda, with a small number conducted in English, depending on the preferences of the participants.

### Assessments

#### Psychiatric evaluations

Psychiatric illness was diagnosed using the Mini International Neuropsychiatric Interview, a short diagnostic instrument that has undergone validation against the Structured Clinical Interview for the DSM-IV–TR Axis I and the Composite International Diagnostic Interview and takes 15 min to administer.^25^ Validation studies have been conducted in African settings such as Morocco, and the instrument has widely been used in other parts of Africa including Uganda.^13,26,27^

The severity of psychosis was determined using the Young Mania Rating Scale (YMRS),^28^ the Brief Psychiatric Rating Scale (BPRS) ^28,29^ and the Patient Health Questionnaire (PHQ-9)^30^ with respect to the primary diagnosis. Items on these tools are not mutually exclusive; a participant can have more than one characteristic on the same tool. The YMRS is a clinician-administered tool that is widely used to assess the severity of manic symptoms on the basis of 11 items, including elevated mood, irritability and aggressive behaviour. Each item is scored from 0 to 4 on a Likert scale; a total score is obtained by summing individual item scores. The YMRS is a reliable and valid measure of manic symptoms that is used in both clinical and research settings.^28^ The PHQ-9 is a common method for screening and measuring depression. It asks about symptoms an individual may have experienced in the past 2 weeks, based on the criteria for major depressive disorder. Scores range from 0 to 27, with severity grades of 5–9 (mild); 10–14 (moderate); 15–19 (moderately severe) and 20–27 (severe). The BPRS is widely used to evaluate the severity of psychiatric symptoms, particularly in individuals with psychosis or schizophrenia spectrum disorders. It consists of 18 items including hallucinations, delusions and blunted affect and is rated on a Likert scale ranging from 1 to 7. Total scores range between 18 and 126. Its brevity, ease of administration and reliability make it a preferred instrument for assessing psychiatric symptoms in both clinical and research settings. Cognitive function was assessed using the International HIV Dementia Scale.^31^ The Mini-Mental State Examination (MMSE) was used to grade levels of cognitive function. This scale, consisting of 11 items, was created by Folstein et al^32^ and has a total score of 30 points. Administration typically requires approximately 10 min.

#### Laboratory examinations

HIV testing was conducted as part of standard clinical investigations following admission. A DETERMINE I/II test kit^33^ was used for initial screening, and the results were confirmed using STAT PAK^34^ and UNIGOLD test kits.^35^ Comprehensive pre- and post-test counselling sessions were provided to all study participants. Other laboratory assessments included a complete blood count, CD4 cell count and VDRL test for syphilis.

HIV-positive individuals were graded on the WHO clinical scale for stage of HIV disease. This chart was developed for the surveillance of HIV/AIDS to track temporal, geographic and risk-group patterns while also estimating the prevalence of HIV/AIDS-related illnesses. Surveillance definitions were initially introduced in 1982; since then, various definitions have been used for reporting purposes at both national and international levels.^17^ Individuals who tested positive for HIV and satisfied the eligibility criteria for commencing antiretroviral therapy were referred to HIV treatment clinics situated at either Mulago or Butabika hospitals.

## Data analysis

We calculated means and medians as applicable and employed the Mann–Whitney U test to compare scores related to performance and severity on psychiatric scales between these two groups. We also conducted bivariate analyses using 2 × 2 tables to explore the associations between disease symptoms and HIV status, with stratification by diagnosis. The data included scores related to a particular symptom, divided into the following categories: mild, moderate and severe. Comparisons between HIV positive and HIV negative were made only for symptoms considered to be moderate or severe. The analyses were performed with the assumption that when using the Pearson χ^2^ test, each cell would exceed five counts. In instances where the expected counts were less than five, Fisher's exact test was used instead. We used a logistic regression model to calculate odds ratios for the manifestation of these symptoms in both HIV-positive and HIV-negative individuals. For each psychotic disorder and symptom, multivariable models were developed using a forward stepwise approach, while accounting for HIV status, gender, age and education level. The data analysis was conducted using STATA version 10 (StataCorp, College Station, TX, USA) with 95% confidence intervals.

## Results

There were 478 patients enrolled in the study, comprising 202 males and 276 females. The overall mean age was 30.7 years. The HIV positive group had a higher mean age compared with the HIV negative group (33.2 years *v.* 29.6 years, *P* < 0.001). Although the study group had more females compared with males (58% *v.* 42%), there was a significantly higher proportion of females compared with males in the HIV-positive group (72% *v.* 28%, *P* < 0.001). The average CD4 count in the HIV positive group was 309 cells/μL. As shown in Table 1, HIV-negative individuals were more likely to be single (60%) and reported more history of previous mental illness, with a median of two episodes.

**Table 1:** Demographic and clinical characteristics of individuals with psychosis

Characteristic	HIV+ (*n* = 156) *n* (%)	HIV− (*n* = 322) *n* (%)
**Gender**		
Male	44 (28.21)	158 (49.07)
Female	112 (71.79)	164 (50.93)
**Age, years**		
18–25	31 (19.87)	124 (38.51)
26–30	36 (23.08)	77 (23.91)
31–40	60 (38.46)	87 (27.02)
41–60	29 (18.59)	34 (10.56)
**Marital status**		
Single	69 (44.23)	192 (59.63)
Married/cohabiting	47 (30.13)	75 (23.29)
Separated/divorced	40 (25.64)	55 (17.08)
**Family history of mental illness**		
Yes	57 (36.54)	152 (47.20)
Past episodes of mental illness median (IQR, max)	1 (2, 11)	2 (4, 11)
CD4 cells/μL (mean, s.d.)	309 (238)	
**WHO clinical stage**^a^		
1	31 (19.87)	
2	49 (31.41)	
3	52 (33.33)	
4	7 (4.49)	

IQR, interquartile range; max, maximum; WHO, World Health Organization.

a. This was determined for only 139 participants.

Almost two-thirds (64%) of the HIV patients with psychosis presented in WHO clinical stages 2 and 3, whereas nearly a quarter (24%) were in stages 1 and 4. The HIV-positive participants had worse cognitive test scores on the MMSE compared with the HIV-negative individuals, as reported by Nakasujja et al.^36^

Notably, psychotic disorder not otherwise specified was more than ten times more prevalent in the HIV-positive group, albeit with a relatively small number of cases. Schizophrenia was observed less frequently among individuals who tested positive for HIV (Table 2).

**Table 2:** Mini International Neuropsychiatric Interview diagnoses among HIV-positive and HIV-negative individuals

	HIV+ *n* (%)	HIV− *n* (%)	*χ*^2^	*P*-value
**Diagnosis**				
Mania with psychotic features	103 (66.02)	209 (64.91)	0.06	0.810
Psychosis not otherwise specified	14 (8.97)	2 (0.62)	22.67	0.000
Schizophrenia	20 (12.82)	80 (24.84)	9.18	0.002
Major depression with psychotic features	21 (13.46)	34 (10.56)	0.85	0.351

**Fig. 1:**
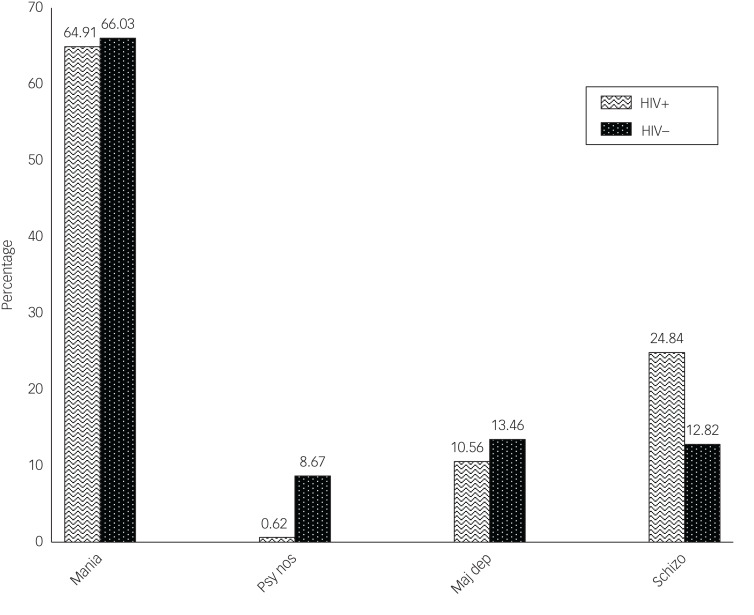
Types of psychosis by HIV status among study participants. Mania, mania with psychotic features; psy nos, psychotic disorder not otherwise specified; maj dep, major depression with psychotic features; schizo, schizophrenia.

**Fig. 2:**
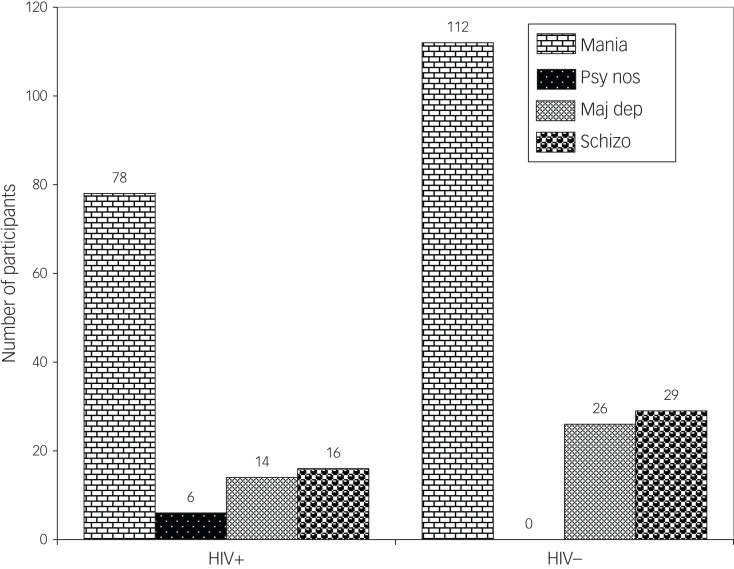
Types of psychosis among female participants by HIV status. Mania, mania with psychotic features; psy nos, psychotic disorder not otherwise specified; maj dep, major depression with psychotic features; schizo, schizophrenia.

**Fig. 3:**
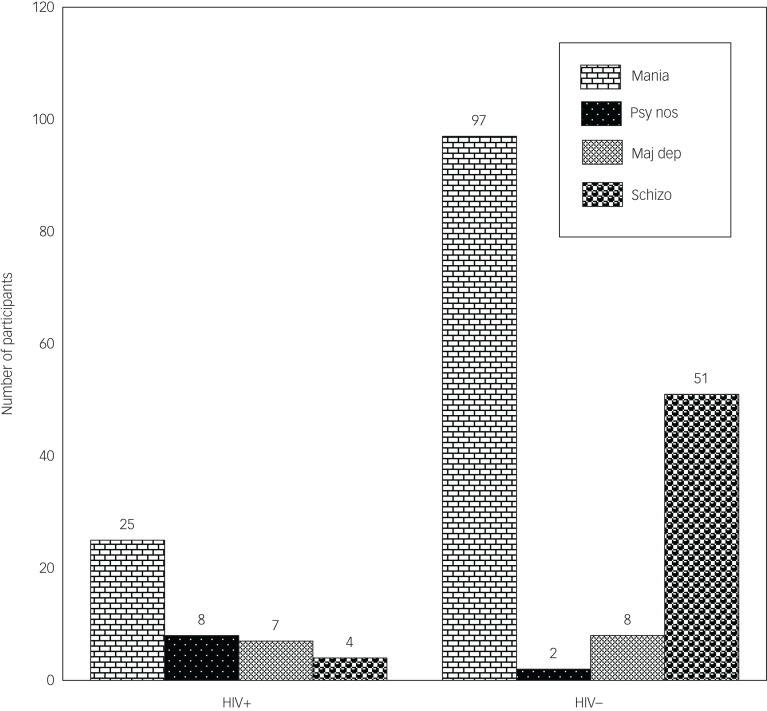
Types of psychosis among male participants by HIV status. Mania, mania with psychotic features; psy nos, psychotic disorder not otherwise specified; maj dep, major depression with psychotic features; schizo, schizophrenia.

**Fig. 4:**
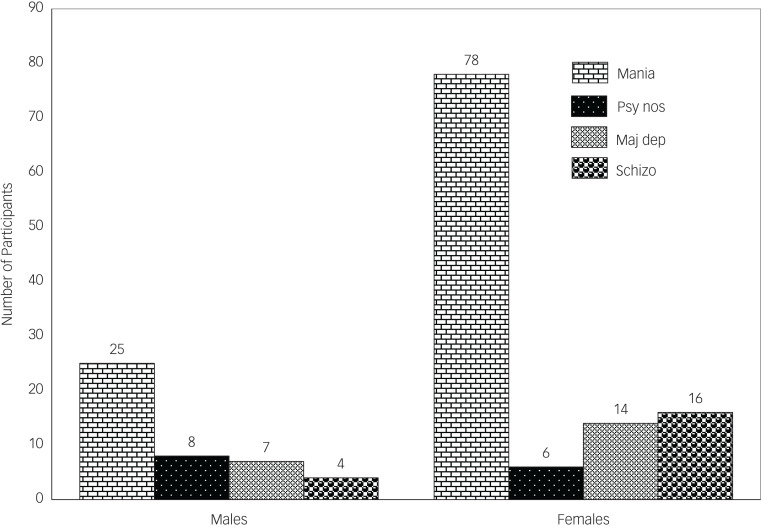
Types of psychosis by sex among HIV-positive participants. Mania, mania with psychotic features; psy nos, psychotic disorder not otherwise specified; maj dep, major depression with psychotic features; schizo, schizophrenia.

There were similar presentations of psychoses in HIV-positive and HIV-negative individuals, except in those with schizophrenia or psychosis not otherwise specified (Fig. 1). Females who were HIV positive had a higher proportion of mania with psychotic features compared with their male counterparts (Figs. 2 and 4). Similarly, in both males and females who were HIV positive, mania was the most common diagnosis, but this was also the case for the HIV-negative participants (Figs. 1–4).

We found statistically significant differences between HIV-positive and HIV-negative individuals with respect to BPRS scores. Specifically, a higher proportion of HIV-negative individuals exhibited mannerisms and posturing (19.88% *v.* 7.69%, *P* = 0.001), grandiosity (49.38% *v.* 37.18%, *P* = 0.012), unusual thoughts (36.34% *v.* 24.36%, *P* = 0.009) and blunted affect (30.12% *v.* 17.95%, *P* = 0.005); see Table 3 for details.

**Table 3:** Comparison of psychotic symptoms among HIV-positive and HIV-negative people as measured with the Brief Psychiatric Rating Scale

Clinical characteristic	HIV+ *n* (%)	HIV− *n* (%)	*χ*^2^	*P*-value
Somatic concern	73 (46.79)	121 (37.58)	3.70	0.054
Anxiety	82 (52.56)	159 (49.38)	0.43	0.514
Emotional withdrawal	49 (31.41)	116 (36.02)	0.99	0.320
Concept disorganisation	45 (28.85)	118 (36.65)	2.85	0.092
Feelings of guilt	60 (38.46)	101 (31.37)	2.37	0.124
Tension	49 (31.41)	96 (29.81)	0.13	0.723
Mannerisms and posturing	12 (7.69)	64 (19.88)	11.67	0.001
Grandiosity	58 (37.18)	159 (49.38)	6.31	0.012
Depressed mood	52 (33.33)	100 (31.06)	0.25	0.616
Hostility	49 (31.41)	116 (36.02)	0.99	0.320
Suspiciousness	63 (40.38)	152 (47.20)	1.98	0.160
Hallucinatory	75 (48.08)	149 (46.27)	0.14	0.711
Motor retardation	50 (32.05)	113 (35.09)	0.43	0.511
Uncooperativeness	42 (26.92)	102 (31.68)	1.13	0.288
Unusual thought	38 (24.36)	117 (36.34)	6.89	0.009
Blunted affect	28 (17.95)	97 (30.12)	8.07	0.005
Excitement	65 (41.67)	142 (44.10)	0.25	0.615
Disorientation	72 (46.15)	125 (38.82)	2.33	0.127

Table 4 shows a comparison of the clinical characteristics of HIV-positive and HIV-negative individuals with various psychoses. HIV-negative participants with mania with psychotic features had notably higher scores for increased motor activity compared with their HIV-positive counterparts (30.62% *v.* 17.48%, *P* = 0.013). In addition, a significantly higher proportion of HIV-positive participants exhibited irritability compared with the HIV-negative group (*P* = 0.031). Furthermore, 22.33% of participants with a language disorder were HIV positive, compared with 8.61% who were HIV negative (*P* = 0.001), and more unkempt individuals were found among the HIV-positive participants compared with the HIV-negative ones (*P* = 0.034). Insight was significantly lacking in HIV-positive individuals than HIV-negative individuals (*P* < 0.001). These findings are detailed in Table 4.

**Table 4:** Clinical characteristics among HIV-positive and HIV-negative individuals for each diagnosis

	HIV+ *n* (%)	HIV− *n* (%)	*χ*^2^	*P*-value
**Clinical characteristics**				
**YMRS in mania**^a^				
Elevated mood	19 (18.45)	35 (16.75)	0.14	0.709
Motor activity	18 (17.48)	64 (30.62)	6.15	0.013
Sexual interest	8 (7.77)	14 (6.70)	0.12	0.729
Sleep	41 (39.81)	86 (41.15)	0.05	0.820
Irritability	11 (10.68)	9 (4.31)	4.67	0.031
Speech	6 (5.83)	11 (5.26)	0.04	0.837
Language disorder	23 (22.33)	18 (8.61)	11.26	0.001
Content of thought	9 (8.74)	21 (10.05)	0.14	0.712
Disruptive behaviour	3 (2.91)	5 (2.39)	0.07	0.785
Appearance	26 (25.24)	32 (15.31)	4.47	0.034
Lack of Insight	45 (43.69)	30 (14.35)	32.52	0.000
**BPRS in psy nos**^b^				
Somatic concern	10 (71.42)	1 (50.00)		0.567
Anxiety	9 (64.29)	1 (50.00)		0.568
Emotional withdrawal	5 (35.71)	0 (0.00)		1.000
Concept disorganisation	6 (42.86)	0 (0.00)		1.000
Feelings of guilt	6 (42.86)	0 (0.00)		1.000
Tension	8 (57.14)	1 (50.00)		1.000
Grandiosity	4 (28.57)	1 (50.00)		0.433
Depressed mood	5 (35.71)	0 (0.00)		1.000
Hostility	5 (35.71)	1 (50.00)		1.000
Suspiciousness	3 (21.43)	1 (50.00)		1.000
Hallucinatory	7 (50.00)	1 (50.00)		1.000
Motor retardation	7 (50.00)	1 (50.00)		1.000
Uncooperativeness	2 (14.29)	1 (50.00)		0.625
Unusual thought	2 (14.29)	1 (50.00)		0.625
Blunted affect	4 (28.57)	1 (50.00)		1.000
Excitement	6 (42.86)	1 (50.00)		1.000
Disorientation	6 (42.86)	2 (100.00)		0.150
**BPRS in schizophrenia**				
Somatic concern	11 (55.00)	43 (53.75)	0.01	0.911
Anxiety	14 (70.00)	52 (65.00)	0.18	0.673
Emotional withdrawal	15 (75.00)	64 (80.00)	0.24	0.623
Concept disorganisation	8 (40.00)	53 (66.25)	4.63	0.031
Feelings of guilt	7 (35.00)	35 (43.75)	0.50	0.478
Tension	9 (45.00)	29 (36.35)	0.52	0.471
Mannerisms and posturing	5 (25.00)	44 (55.00)	5.76	0.016
Grandiosity	3 (15.00)	28 (35.00)	2.99	0.084
Depressed mood	10 (50.00)	36 (45.00)	0.16	0.688
Hostility	7 (35.00)	28 (35.00)	3.85	0.999
Suspiciousness	11 (55.55)	56 (70.00)	1.63	0.202
Hallucinatory	15 (75.00)	63 (78.75)	0.13	0.717
Motor retardation	6 (30.00)	45 (56.25)	4.41	0.036
Uncooperativeness	5 (25.00)	30 (37.50)	1.10	0.295
Unusual thought	11 (55.00)	58 (72.50)	2.29	0.130
Blunted affect	10 (50.00)	63 (78.75)	6.71	0.009
Excitement	6 (30.00)	25 (31.25)	0.01	0.914
Disorientation	14 (70.00)	38 (47.50)	3.25	0.072
**PHQ-9 in depression**				
Little interest	14 (66.67)	21 (61.76)	0.13	0.714
Feeling down	16 (76.19)	21 (61.76)	1.23	0.268
Trouble sleeping	13 (61.90)	22 (64.71)	0.04	0.834
Feeling tired	15 (71.43)	22 (59.71)	0.27	0.606
Poor appetite	14 (66.67)	19 (55.88)	0.63	0.428
Feeling bad	11 (52.38)	19 (55.88)	0.06	0.800
Trouble concentrating	12 (57.14)	19 (55.88)	0.01	0.927
Slow activity	13 (61.90)	23 (67.65)	0.19	0.663
Better dead	8 (38.10)	15 (47.06)	0.19	0.66

YMRS, Young Mania Rating Scale (only individuals with diagnosis of mania with psychotic features were compared); BPRS, Brief Psychotic Rating Scale (only individuals with diagnosed with psychosis not otherwise specified (psy nos) or schizophrenia were compared); PHQ-9, Patient Health Questionnaire 9 (only individuals diagnosed with depression with psychotic features were compared).

a. Characteristics had to be moderate to severe.

b. Fisher's exact test was used.

Among participants with schizophrenia, HIV-positive participants exhibited lower rates of concept disorganisation (40.00% *v.* 66.25%, *P* = 0.019), mannerisms and posturing (25.00% *v.* 55.00%, *P* = 0.016), motor retardation (30.00% *v.* 56.25%, *P* = 0.036) and blunted affect (50.00% *v.* 78.75%, *P* = 0.009) compared to their HIV-negative counterparts (Table 4). All chi-squared analyses had one degree of freedom. The median score for mania symptoms was higher in the HIV-positive group; however, this difference was not significant based on the Mann–Whitney test score (*P* = 0.065; Table 5).

**Table 5:** Severity of psychiatric illness in the HIV-positive and HIV-negative groups

Rating scale	HIV+	HIV−	*P*-value^a^	U test	*R*^2^	Adjusted *R*^2^
YMRS^b^ Median (IQR)	17 (10–24)	15 (7–22)	0.065	1.791	0.0100	0.0077
PHQ-9^c^ Median (IQR)	10 (7–16)	10 (5–15)	0.506	7.121	0.0603	0.0581
BPRS Median (IQR)	14 (5–22)	14 (8–27)	0.205	−1.308	0.0056	0.0031

YMRS, Young Mania Rating Scale; IQR, interquartile range; PHQ-9, Patient Health Questionnaire 9; BPRS, Brief Psychotic Rating Scale.

a. Value was determined using Mann–Whitney test.

b. Value determined only for patients with mania with psychotic features.

c. Value determined only for patients with depression with psychotic features.

In the linear regression analysis, each unit increase in the YMRS score was associated with being HIV positive (β = 4.17, 95% CI 1.96–6.38, *P* < 0.001), whereas age > 30 years (β = −2.19, 95% CI −4.25 to −0.13, *P* = 0.037) and female gender (β = −6.04, 95% CI −8.14 to –3.94, *P* = <0.001) were associated with decreased scores. On the BPRS, being female was associated with decreased scores (β = −8.15, 95% CI −10.61 to −5.69, *P* < 0.001), whereas PHQ scores were increased for HIV-positive individuals (β = 2.35, 95% CI 1.44–3.25, *P* < 0.001). There was no significant relationship between WHO stage and BPRS score (β = −1.52, 95% CI −2.58 to 5.63, *P* = 0.464). The HIV-positive individuals were 2.6 times more likely (odds ratio 2.62 95% CI 1.69–4.06) to be cognitively impaired on the MMSE compared with the HIV-negative group.

The HIV-positive participants were more likely to have the following characteristics according to the logistic regression: motor activity (odds ratio 0.55, 95% CI 0.32–0.94, *P* = 0.030), irritability (odds ratio 3.07, 95% CI 1.33–7.09, *P* = 0.008), emotional withdrawal (odds ratio 0.34, 95% CI 0.18–0.66, *P* = 0.001), impaired content of thought (odds ratio 2.39, 95% CI 1.35–4.21, *P* = 0.003), feelings of guilt (odds ratio 2.92, 95% CI 1.55–5.59, *P* = 0.001), mannerisms and posturing (odds ratio 0.19, 95% CI 0.06–0.64, *P* = 0.007), grandiosity (odds ratio 0.51, 95% CI 0.31–0.86, *P* = 0.011), suspiciousness (odds ratio 0.45, 95% CI 0.24–0.85, *P* = 0.015), blunted affect (odds ratio 0.22, 95% CI 0.10–0.49, *P* < 0.001), excitement (odds ratio 0.51, 95% CI 0.31–0.85, *P* = 0.001) and disorientation (odds ratio 2.94, 95% CI 1.51–5.72, *P* = 0.001). In the multivariable analysis (Table 6), HIV positivity was associated with schizophrenia (odds ratio 0.46, 95% CI 0.26–0.80, *P* = 0.006), whereas female gender was associated with major depression with psychotic features (odds ratio 2.05, 95% CI 1.07–3.85, *P* = 0.028), psychotic disorder not otherwise specified (odds ratio 0.18, 95% CI 0.05–0.57, *P* = 0.003) and schizophrenia (odds ratio 0.59, 95% CI 0.37–0.94, *P* = 0.027).

**Table 6:** Multivariable analysis of psychotic symptoms and HIV seropositivity

	Adjusted odds ratio (95% CI)	*P*-value
**Clinical characteristics**		
**YMRS in mania**^a^		
Elevated mood	1.24 (0.69–2.20)	0.471
Motor activity	0.55 (0.32–0.94)	0.03
Sleep	0.94 (0.62–1.43)	0.779
Irritability	3.08 (1.33–7.09)	0.008
Speech	2.08 (0.29–14.89)	0.467
Language disorder	1.03 (0.41–2.61)	0.944
Content of thought	2.93 (1.35–4.22)	0.003
Disruptive behaviour	1.03 (0.49–2.19)	0.292
Appearance	1.24 (0.29–5.27)	0.768
Lack of insight	1.47 (0.88–2.44)	0.140
**BPRS**		
Somatic concern	1.25 (0.65–2.39)	0.503
Emotional withdrawal	0.34 (0.18–0.66)	0.001
Concept disorganisation	0.74 (0.39–1.40)	0.353
Feelings of guilt	2.92 (1.53–5.59)	0.001
Tension	1.17 (0.59–2.32)	0.651
Mannerisms	0.19 (0.06–0.64)	0.007
Grandiosity	0.51 (0.31–0.86)	0.011
Depressed mood	0.76 (0.40–1.45)	0.405
Hostility	0.64 (0.32–1.31)	0.221
Suspiciousness	0.45 (0.24–0.85)	0.015
Motor retardation	0.62 (0.30–1.30)	0.205
Uncooperativeness	0.88 (0.39–1.96)	0.751
Unusual thought	0.17 (0.06–0.48)	0.001
Blunted affect	0.22 (0.10–0.49)	0.000
Excitement	0.51 (0.31–0.85)	0.009
Disorientation	2.95 (1.52–5.73)	0.001
**PHQ-9 in depression**		
Little interest	1.14 (0.73–2.73)	0.303
Feeling down	2.13 (0.95–4.83)	0.068
Trouble sleeping	1.14 (0.61–2.14)	0.683
Feeling tired	1.58 (0.77–3.24)	0.211
Poor appetite	1.61 (0.83–3.09)	0.126
Feeling bad	1.08 (0.60–1.93)	0.806
Trouble concentrating	1.21 (0.67–2.21)	0.526
Slow activity	1.06 (0.56–2.01)	0.850
Better dead	0.98 (0.55–1.75)	0.945

YMRS, Young Mania Rating Scale BPRS, Brief Psychotic Rating Scale; PHQ-9, Patient Health Questionnaire 9.

a. Characteristics had to be moderate to severe.

## Discussion

This study compared clinical characteristics of patients with psychoses among HIV-positive and HIV-negative individuals; the results showed differences between the two groups, consistent with findings of previous research studies.^2,3^ Although some of the symptoms may have been indistinguishable from those of a functional psychosis, there were many differences, as has been alluded to by other studies.^1,37^ In particular, we found that HIV positive individuals tended to have more symptoms consistent with psychotic disorder not otherwise specified. Infections that affect the brain, such as neurosyphilis and other neuro-infections including HIV, could result in inflammatory processes whose effects may be distinct.^38^

There is evidence that the virus attacks the brain tissues early in HIV infection.^39^ This may explain the presence of HIV psychosis in patients in the early stages of HIV disease in this study, indicating that HIV-associated psychosis does not occur only in the late stages of HIV as has previously been suggested.^37^ The HIV-positive individuals tended to be older, and, as typically seen in HIV clinic settings, there was a greater representation of females in this group.^40,41^ HIV affects this gender more for a variety of reasons, including higher rates of infection in the females owing to lack of emancipation, bargaining power for sex, and physiological and anatomical differences.^40,42^ In addition, females have a greater tendency to seek healthcare.^43^

HIV-associated psychosis is an established phenomenon that results as a neuropsychiatric complication of HIV infection.^37^ Our understanding of the mechanism by which this occurs has been advanced through various theories that implicate direct invasion of brain tissue by the virus, resulting in toxin production.^39,44^ The glutamate–*N*-methyl-d-aspartic-acid (NMDA)–calcium pathway is one such mechanism of toxin production. Blockage of calcium influx at the NMDA receptor of a cell inhibits glutamate activation for synaptic potentiation, resulting in psychosis. Substances that block this receptor include quinolinic acid, an NMDA agonist, which is released from macrophages when induced by HIV proteins. In high concentrations, as may be found in the central nervous systems of HIV-positive individuals, the acid is neurotoxic.^45^ HIV-positive patients also have increased concentrations of kynurenic acid, an endogenous NMDA antagonist. However, levels are higher in those who have both psychosis and HIV.^46^ Similarly, patients with schizophrenia also have increased cerebrospinal fluid levels of kynurenic acid,^47^ with further increases in those who are also HIV positive.^48^ Psychosocial theories implicate HIV as a predisposing factor for psychosis, either because of the psychological stress of having a stigmatised chronic disease^49^ or through HIV-related neuropathological mechanisms, as may occur if an individual has opportunistic infections.^50,51^ Given this difference in aetiology, a number of patients with HIV may present with characteristics that do not fit the classical primary psychiatric diagnoses. This was clearly manifested here by the HIV-positive group showing a significant representation of psychosis not otherwise specified, in a clear deviation from the primary DSM-V^6^ and ICD diagnostic criteria.^25^

Our observation of irritability in mania with psychotic features being positively associated with HIV seropositivity parallels the findings of Nakimuli et al and is further supported by the work of Ellen et al, who identified a similar trend, especially in the immunologically suppressed.^13,52^ We also found that heightened motor activity was negatively associated with HIV-positive participants in the mania with psychotic features category; this finding was again similar to that of Nakimuli et al.^13^ There are limited data on the clinical characteristics of HIV-related mania. Schizophrenia has not been directly linked to HIV, and a handful of studies have found no association.^53^ However, in this study, schizophrenia was more prevalent in the HIV-negative participants than the HIV-positive ones, analogous to the findings of other studies.^22^

We observed negative associations of HIV seropositivity with emotional withdrawal, mannerisms and posturing, grandiosity, suspiciousness, unusual thoughts, blunted affect and excitement. Conversely, feelings of guilt and disorientation were positively associated with HIV seropositivity. The increased presence of guilt may have been due to the stigma and internalised negativity often experienced by people living with HIV.^43^ Disorientation could be linked to the cognitive impairments frequently observed in HIV-associated neurocognitive disorders.^36^ These contrasting findings suggest that the presentation of psychosis may differ based on HIV status, with HIV-positive individuals potentially exhibiting a distinct form of the disorder. This underscores the importance of considering HIV status during clinical evaluation and management of patients with psychosis. Our findings offer novel insights into the clinical presentation of psychosis in individuals with HIV and highlight the need for further research in this understudied area owing to the limited existing data.

This study had limitations; for instance, the onset of the psychosis in relation to the time that the individuals became HIV positive was difficult to determine precisely in many instances. However, we collected information from patient files and also made estimates of previous episodes of mental illness. No neuro-imaging studies were conducted in this work to exclude the occurrence of other neurological conditions that could cause psychosis. However, all patients underwent a physical examination and laboratory tests for common opportunistic infections such as toxoplasmosis and cryptococcal meningitis. No multiplicity correction was done. Nonetheless, we used tools with the rigour to detect different presentations among the participants.

### Interpretation of findings

The effects of HIV on the brain results in specific insults that manifest in distinct ways.^5,37^ HIV-related psychosis can occur at any stage of HIV infection, although it is more common in the later stages of disease, especially if antiretroviral treatment is not initiated early enough.^2^ Many individuals still present late for treatment in Uganda, despite the UNAIDS targets of 90/90/90.^54^ It certainly remains pertinent that clinicians are able to detect HIV-related psychosis. Hence, better understanding of the clinical presentation of psychosis, as shown by this study, remains a key factor in serving the HIV-positive population, given their unique presentation and response to treatments for psychosis.

## Data Availability

The data that support the findings of this study are available on request from the corresponding author, N.N.
